# Description of the SAGhE Cohort: A Large European Study of Mortality and Cancer Incidence Risks after Childhood Treatment with Recombinant Growth Hormone

**DOI:** 10.1159/000435856

**Published:** 2015-07-23

**Authors:** Anthony J Swerdlow, Rosi Cooke, Kersti Albertsson-Wikland, Birgi Borgström, Gar Butler, Stefan Cianfarani, Pete Clayton, Joë Coste, Annalis Deodati, Emmanue Ecosse, Rut Gausche, Claudi Giacomozzi, Wielan Kiess, Anita C.S Hokken-Koelega, Claudia E Kuehni, Fabienn Landier, Mar Maes, Primus-E Mullis, Rolan Pfaffle, Lar Sävendahl, Gri Sommer, Murie Thomas, Sall Tollerfield, Gladys R.J Zandwijken, Jean-Claud Carel

**Affiliations:** ^a^Division of Genetics and Epidemiology, Institute of Cancer Research, London, UK; ^b^Division of Breast Cancer Research, Institute of Cancer Research, London, UK; ^c^Institute of Neuroscience, Department of Physiology/Endocrinology, Sahlgrenska Academy, University of Gothenburg, Gothenburg, Sweden; ^d^Department of Clinical Science, Intervention, and Technology, Karolinska Institutet, Stockholm, Sweden; ^e^UCL Institute of Child Health, London, UK; ^f^University College London Hospitals NHS Foundation Trust, London, UK; ^g^Dipartimento Pediatrico Universitario Ospedaliero ‘Bambino Gesù’ Children's Hospital, Tor Vergata University, Rome, Italy; ^h^Department of Women's and Children's Health, Karolinska Institutet, Stockholm, Sweden; ^i^Royal Manchester Children's Hospital, Central Manchester University Hospitals NHS Foundation Trust, Manchester Academic Health Science Centre, Manchester, UK; ^j^Centre for Paediatrics and Child Health, Institute of Human Development, Faculty of Medical and Human Sciences, University of Manchester, Manchester, UK; ^k^Biostatistics and Epidemiology Unit, and Approches Psychologiques et Epidémiologiques des Maladies Chroniques Equipe d'Accueil, Paris, France; ^l^Groupe Hospitalier Cochin-Saint Vincent de Paul and University Paris Descartes, Paris, France; ^m^Hospital for Children and Adolescents and Centre of Pediatric Research, University of Leipzig, Leipzig, Germany; ^n^Dipartimento di Medicina Pediatrica ‘Bambino Gesù’ Children's Hospital, Rome, Italy; ^o^Dutch Growth Research Foundation, Rotterdam, The Netherlands; ^p^Erasmus Medical Center/Sophia Children's Hospital, Rotterdam, The Netherlands; ^q^Institute of Social and Preventive Medicine, University of Bern, Bern, Switzerland; ^r^University Paris Diderot, Sorbonne Paris Cité, Assistance Publique-Hôpitaux de Paris; ^s^Service d'Endocrinologie Diabétologie Pédiatrique et Centre de Référence des Maladies Endocriniennes Rares de la Croissance, Hôpital Robert Debré, Assistance Publique-Hôpitaux de Paris; ^t^Institut National de la Santé et de la Recherche Médicale Unité CIE-5, Paris, France; ^u^Division of Pediatric Endocrinology, Department of Pediatrics, Cliniques Universitaires St. Luc, Université Catholique de Louvain, Brussels, Belgium; ^v^Division of Paediatric Endocrinology, Diabetology and Metabolism, University Children's Hospital Bern, Inselspital, Bern, Switzerland; ^w^Division of Pediatric Endocrinology, Department of Women's and Children's Health, Karolinska Institutet, Stockholm, Sweden; ^x^Belgian Study Group for Pediatric Endocrinology (BSGPE), Brussels, Belgium

**Keywords:** Growth hormone, Cohort, Europe, Cancer, Mortality

## Abstract

**Background:**

The long-term safety of growth hormone treatment is uncertain. Raised risks of death and certain cancers have been reported inconsistently, based on limited data or short-term follow-up by pharmaceutical companies. ***Patients and Methods:*** The SAGhE (Safety and Appropriateness of Growth Hormone Treatments in Europe) study assembled cohorts of patients treated in childhood with recombinant human growth hormone (r-hGH) in 8 European countries since the first use of this treatment in 1984 and followed them for cause-specific mortality and cancer incidence. Expected rates were obtained from national and local general population data. The cohort consisted of 24,232 patients, most commonly treated for isolated growth failure (53%), Turner syndrome (13%) and growth hormone deficiency linked to neoplasia (12%). This paper describes in detail the study design, methods and data collection and discusses the strengths, biases and weaknesses consequent on this.

**Conclusion:**

The SAGhE cohort is the largest and longest follow-up cohort study of growth hormone-treated patients with follow-up and analysis independent of industry. It forms a major resource for investigating cancer and mortality risks in r-hGH patients. The interpretation of SAGhE results, however, will need to take account of the methods of cohort assembly and follow-up in each country.

## Background

Growth hormone (GH) treatment has been used since 1957 to treat GH deficiency, and thereafter increasingly for short stature due to other causes. Initially, hormone extracted from human pituitaries (p-hGH) was used, and then since the mid-1980s synthetic recombinant human GH (r-hGH). Concerns about long-term safety were initially caused by an outbreak of Creutzfeldt-Jakob disease consequent on prion infection of pooled pituitaries used to produce human GH, whose use was therefore discontinued in 1985. Subsequently, concerns were raised by reports of apparent excesses of leukaemia [1,2] and other cancers [3], and more recently of mortality in France [4,5] although not in three other SAGhE countries [6]. There have been a number of studies investigating cancer and mortality risks in such patients [7,8,9,10,11,12,13,14,15,16,17,18,19,20,21,22], but these have limitations and leave considerable uncertainty.

Although cohort studies have been published with tens of thousands of patients [23,24,25], these have been based on pharmaceutical company databases and very short follow-up by physician reporting of adverse events. These databases have produced much valuable information, but when used for cohort analyses, they provide uncertain evidence on short-term effects (because of potential conflict of interest and potential incompleteness of outcome ascertainment) and no evidence about the long term when, for instance, any cancers or other chronic disease risks consequent on treatment might be expected to manifest.

Two cohort studies of patients treated with p-hGH have reported long-term follow-up: one study of 1,848 UK patients followed for an average of 21 years [26] and one of 6,107 US patients followed for an average of 17 years [27]. The dosage and timing of p-hGH treatment, however, is very different from that for r-hGH. The only published cohort studies of r-hGH patients not originating from follow-up in a pharmaceutical company database have been of 360 cancer patients in the US followed for an unspecified period [28] and 289 hypopituitarism patients in Sweden followed for an average of 5 years [29]. The SAGhE (Safety and Appropriateness of Growth Hormone Treatments in Europe) study was therefore initiated in Europe to provide a large-scale international collaborative cohort study of r-hGH-treated patients with long-term follow-up for cancer incidence and mortality conducted independently of pharmaceutical companies.

This paper describes the rationale, design, recruitment and methods of the SAGhE cohort study and discusses its strengths and weaknesses. As well as conducting the cohort study of risks of mortality and cancer incidence described here, the SAGhE project also investigated height and quality of life: the methods and interpretation of these components of SAGhE will be presented elsewhere.

## Patients and Methods

The study was of cohort design, conducted in 8 European countries, with the design and conduct coordinated from the outset and analyses centralised. The countries and data sources are shown in table 1. In brief, with appropriate ethics and privacy committee approvals, we attempted to identify in each country all resident patients who were born before 1991-1995 (the exact year depending on the country), who had been treated with r-hGH at paediatric endocrine clinics at any time up to a date during 2007-2009, depending on the country (or in France and Sweden up to 1997) (table 2), and who had never been treated with p-hGH. In Sweden and Germany, about 30% of the patients had been treated in clinical trials of GH, in France 10.5%, in Belgium and the Netherlands about 7% or less, and in Italy, Switzerland, and the UK very few or none. In several countries (Belgium, Germany, Italy, the Netherlands, and the UK), the small proportion of patients receiving r-hGH because of chronic renal failure were sometimes or always registered in a different database from those treated for all other reasons, and identification of the renal patients was more sporadic and less complete than that of other patients. In Germany, national population-based identification was not practical, and identification was instead limited to patients meeting the above criteria from selected clinics. In Switzerland, ascertainment was clinic-based but covering most of the country, and in Italy, ascertainment was population-based in part of the country and clinic-based elsewhere. In Scotland, one clinic (Dundee) was not included, but identification was otherwise population-based. In each country, the principle was followed that patients were included if ascertainment was either prospective (i.e. patients were identified as they started treatment) or if retrospective identification was close to 100% complete, so that recruitment could not be biased by the subsequent occurrence of cancers, deaths, etc. in cohort members.

In two countries, Germany and the UK, some patients included in the cohort were identified from pharmaceutical company post-marketing surveillance registers. In Germany, these were registers of several companies (Lilly, Pfizer, Merck, Ferring, and Novo Nordisk). In the UK, they were registers run by Kabi Pharmacia (now Pfizer), into which the treating physicians at all centres except one (Cardiff) had entered details of all patients treated at their centre, irrespective of the brand of r-hGH used for treatment. In both countries the registers were compiled at the time the patients started on GH, and hence before the study outcomes occurred; therefore, patient inclusion would not have been biased by outcome.

Data were then extracted from existing databases and case notes (table 3) on demographic variables, parental heights, birth characteristics, results of GH testing and additional endocrine deficiencies, and treatment of those deficiencies, at regular intervals of height, weight, pubertal status, bone age, GH dose and number of weekly injections and associated treatments that might interfere with growth (sex steroids, GnRH agonists, steroids), the use of cranial or total body irradiation, the diagnosis for which r-hGH was prescribed, and, in Belgium, France, Germany, the Netherlands, Sweden, and Switzerland, cancers and deaths occurring during paediatric endocrine follow-up. Ascertained subjects were then excluded from the cohort if their age at the start of treatment was outside the study criteria, or if their sex, date of birth or date of starting treatment or ending follow-up was unknown, or if they were treated at centres where retrospective identification of cases was substantially incomplete or follow-up was not possible.

Follow-up of cohort members for incident cancers, deaths, emigrations, and other losses to follow-up was conducted by various methods depending on the country (tables 4, 5). In Belgium, the Netherlands, Sweden, and the UK, there are national population-based registries for vital status, follow-up, and cancer from which these data were obtained by record linkage. In the other countries, where such databases were not available, a range of sources were used including municipal registries, a national health insurance register, and questionnaires, as detailed in tables 4 and 5. In Belgium, Switzerland, and the UK, all patients followed for mortality were also followed for cancer incidence except small numbers who did not reach the cancer incidence follow-up period. In the Netherlands and Sweden, the situation was similar except for a small proportion of subjects who declined to be included. In the other countries, only subsets of subjects were included in cancer incidence follow-up (table 5): in Germany only patients at one centre were included; in France only patients whose initial diagnosis leading to GH treatment was not cancer, renal disease or certain other diseases (listed in table 5) were included; and in Italy only those who returned questionnaires were included.

Reported cancer diagnoses were validated by cancer registry data or from pathology reports, except in France where they were based on hospital records or coherence of other sources, and in Italy, where no validation was possible. In Belgium, Germany, the Netherlands, Sweden, Switzerland, and the UK, reported second cancers in patients whose initial diagnosis leading to GH treatment was cancer were rechecked for the SAGhE study to ensure that they were not restatements, recurrences or metastases of the original cancer. Because in Italy the ascertainment of cancer was retrospective and potentially highly biased, and there was no source of medical validation of reported diagnoses, we will not include Italian subjects in cancer risk analyses; the numbers of Italian cancers by site will, however, be shown descriptively.

Information about the cause of death was obtained from registries based on death certificates in most countries, supplemented by clinical notes in France and Germany, and was solely taken from clinical notes in the Netherlands.

National cause-specific mortality data and population counts for the general population, to derive ‘expectations’ for mortality rates in the cohort, were obtained from death certificate-based statistics from national statistics offices. National site-specific cancer incidence data, likewise, were obtained from national cancer registries (the Netherlands, Sweden, UK, and Belgium from 2004 onwards) or, where national cancer registration did not exist, from national estimates based on regional registry data (Belgium before 2004, France, Germany, and Switzerland). The degree of diagnostic detail available, for mortality and for cancer incidence statistics, varied between countries. Furthermore, non-malignant meningiomas (which constitute most meningiomas) were not included in German and French national cancer incidence statistics, and were not included in cancer incidence data for the Swiss r-hGH-treated cohort, so our analyses for meningioma incidence will exclude these countries.

Person-years at risk of death will be calculated for each patient by sex, 5-year age-group, single calendar year, and country, starting from the date of the first treatment with r-hGH and ending at whichever occurred earliest of death, loss to follow-up, or a fixed end date for each country (defined on the basis of the point to which follow-up in that country was considered complete at the time the follow-up data were obtained) (table 2). Similar calculations will be conducted for cancer incidence, but with the date of cancer diagnosis as an additional end date for follow-up, and with a fixed end date for each country specific to the cancer incidence analyses (table 2). In Switzerland, cancer incidence follow-up will be censored at age 16 or 21, depending on the canton, because the national cancer registry used to ascertain cancers occurring in the cohort only covered these ages.

Observed numbers of cancers and deaths in the cohort will then be compared with expectations based on application of sex-, age-, country- and year-specific rates in the general population of each country to the person-years at risk in these categories in the cohort, to derive standardised mortality ratios and standardised incidence ratios. Absolute excess rates will be calculated by subtracting the expected from the observed numbers of cases, dividing by person-years at risk and multiplying by 10,000.

Cancer and mortality risks will be analysed in the cohort overall and subdivided by dose and duration of GH treatment and by initial diagnosis for which patients received r-hGH. However, because the initial diagnosis of these patients heavily influences their future mortality and morbidity, and there were many different initial diagnoses, often with few cases per diagnosis, we devised 10 categories of initial diagnosis leading to GH treatment and 4 larger prognostically based groupings of initial diagnosis, which we will use in diagnosis-based analyses requiring larger numbers. The 10 groups are shown in table 6. The 4 larger groups are Ia: isolated growth failure including isolated GH deficiency and idiopathic short stature, Ib: short stature in children born small for gestational age, II: multiple pituitary hormone deficiency, children with conditions/malformations associated with low or modest effects on mortality, chromosomal or syndromic conditions, benign pituitary tumours, and III: high mortality conditions, including malignancy, chronic renal failure, and chromosomal abnormalities at known high risk of cancer. Details are given in the online supplementary material (see www.karger.com/doi/10.1159/000435856). These groupings were based on published evidence and clinical judgments about the prognosis of the individual diagnoses in general, not in relation to GH treatment, and blind to the actual mortality in the study or the outcomes of individual study subjects.

## Cohort Descriptive Variables

A total of 27,550 patients were identified who were treated with r-hGH in the study countries during the study period but not with p-hGH previously (table 7). Twenty six of these were excluded from the cohort under analysis because they were first treated beyond the age of 19 years, 61 were excluded because their date of birth, sex or date of first treatment was unknown, and 21 were excluded because their date of ending follow-up was unknown. In addition, in Germany, patients (n = 19) from 3 centres were omitted because retrospective identification was only achieved for a small proportion of subjects from these centres; in the UK, 720 patients were omitted because of lack of permission for follow-up or because the original anonymised database that had recorded the patients contained insufficient information about these individuals to identify them for follow-up; and in Italy, 2,471 patients were excluded because follow-up in that country was only possible from January 1, 1999 onward, and these patients had no follow-up beyond that date.

This left 24,232 patients, 13,425 male and 10,807 female, who formed the study cohort for mortality analyses. For cancer incidence follow-up, a further 3,756 patients were excluded because of lack of permission or unavailability of data, leaving 20,476 patients, 11,108 male and 9,368 female, as the study cohort. Although these individuals will be included in the overall analyses, individuals who are missing data on more detailed variables, e.g. dose of GH, will be omitted from analyses of those particular variables. Descriptive characteristics of the cohort are shown in table 7. Most subjects started treatment at ages 10-14 years (51%) or 5-9 years (32%). Half (51%) were treated for isolated growth failure, 14% for Turner syndrome, 13% for GH deficiency linked to neoplasia and 21% for other indications, although the proportions varied considerably by country. Based on provocative GH stimulation tests where available to us, we estimate that 76% of the patients with isolated growth failure had isolated GH deficiency (maximum peak <10 ng/ml) and 24% had idiopathic short stature.

Follow-up for mortality was 96.7% complete, excluding data for Italy where completeness data were not available (table 8). Follow-up for cancer incidence was 98.3% complete, excluding Italy and France where cancer incidence ascertainment could not be conducted systematically.

## Discussion

The published literature on risks of death and cancer incidence in relation to GH treatment is limited. Large cohorts of patients treated in childhood with r-hGH and recorded on pharmaceutical company databases have been followed for short periods [5,6,7], whereas longer follow-up is limited to two cohorts of patients treated with p-hGH [8,9], and two small cohorts of a few hundred r-hGH patients each [10,11].

The SAGhE study has successfully assembled a cohort of r-hGH-treated patients with long-term follow-up on a far larger scale than has previously been reported: >24,000 patients with >400,000 person-years of follow-up, an average of 17.1 years per patient. The combination of data from 8 countries enabled this scale of investigation, which is essential to gain the power to examine long-term risks. Inevitably, however, such an international aggregation involves overcoming complexities consequent on differences in clinical practice, and in the strengths and weaknesses of data, between countries. The initial recruitment of patients was population-based and near 100% complete in Belgium, France, the Netherlands and Sweden. In Switzerland and the UK, recruitment was not quite as complete, but unlikely to be biased. In Switzerland there was near 100% ascertainment of patients at clinics covering about 80% of the country, except that for renal insufficiency and cancer as original diagnoses, the entire country was covered. In the UK, although a high proportion of patients had been recorded prospectively on a population basis, there was a 14% shortfall of patients whose identifying details could not be determined retrospectively for follow-up from the non-identifying codes available on the national database. These losses through non-identifiability were mainly at a few centres, but a comparison of descriptive characteristics and mortality of cohort members at these centres and others in the UK did not suggest that the losses were biased. Mortality rates were very similar in patients from high- and low-loss UK centres.

In two countries, ascertainment of patients was more incomplete, with greater potential for bias. In Germany, identification was from a minority of clinics in the country, but in general these clinics had geographic catchment areas. In Italy, completeness of ascertainment was unknown in all areas except Piedmont, Campania and Rome, and hence the extent of any bias in ascertainment was also unknown.

The validity of the recorded information on the diagnosis leading to GH treatment may also have varied between countries. In several countries (Belgium, Italy, France, the Netherlands, Switzerland, and the UK), prescription of GH was restricted, by regulation and/or reimbursement rules or clinical study protocols, to patients with certain diagnoses, and there was therefore potential for recorded diagnoses to be biased towards those for which GH treatment was permitted. In Germany, the Netherlands, Sweden, and Switzerland, we were able to use other information (e.g. case notes) to re-categorise the diagnoses for SAGhE, blind to outcomes. Interpretation of the results for each country will need to take account of the way in which diagnoses were obtained, and any potential biases in this, in that country.

There was also variation between countries in the balance of diagnoses for which GH was prescribed. In Belgium, the Netherlands and the UK, a particularly large proportion of patients had received GH after cancer. In France and Italy, a particularly large proportion of subjects were reported as having isolated growth failure, and in Sweden there were also a large number with isolated growth failure and skeletal dysplasias because of clinical trials on these. It is notable that in France and Italy, the regulations/reimbursement rules gave an incentive to state this diagnosis for marginal cases, in order to be able to prescribe r-hGH, and the diagnostic labels stated at prescription were not reviewed using other information and re-categorised for the SAGhE study. The proportions of patients treated for chronic renal failure varied between countries, probably reflecting the separate and incomplete identification of such patients in some countries, noted above. There was also variation in the sex ratio of patients – a male excess in most countries, but not in Belgium and the Netherlands where females predominated: this reflected, in part at least, larger proportions of patients with Turner syndrome in these than in most other countries.

The SAGhE countries varied in the years of first treatment, and the years of follow-up, included in the study. This adds complexity to the analyses but does not, in principle, bias them. The cohort included both trial and non-trial patients, and where appropriate consideration will therefore need to be given to the potential differences in outcome that could occur between such patients. Countries varied also in the sources of information on, and likely completeness of follow-up for, vital status and cancer incidence. In Sweden, the UK, the Netherlands, Belgium except for cancer incidence before 2004, and Switzerland except for childhood cancer before 1991, follow-up was via national registries (or in Switzerland a network of municipal registries) known to have virtually complete coverage of the resident population, virtually complete ascertainment of deaths, and high levels of completeness of national cancer registration. Mortality follow-up in Germany is also believed to be complete, by tracking patients across residence registries in each region, but German cancer registration data were only available for the area around Leipzig, and hence cancer incidence analyses were limited to Leipzig patients and were likely to be incomplete if patients moved away from the Leipzig area. French mortality follow-up is over 99% complete, omitting small numbers of deaths abroad. French cancer incidence data were based on several incomplete sources including questionnaire responses, insurance records, and death certificate diagnoses, and were probably appreciably incomplete so will not be included in risk analyses. In Italy, mortality follow-up was solely based on linkage to a national death register, with no method to ascertain emigrations and other losses to follow-up, and uncertainty about completeness of death ascertainment. Italian cancer incidence data, as noted earlier, were incomplete and not validated, so they will not be included in risk analyses.

In addition to potential incompleteness of the follow-up system, countries also differed in the extent to which follow-up needed to be censored when patients were known to have left the population covered by the system, e.g. because they emigrated. Such losses to follow-up before the cutoff end date constituted less than 5% of the cohort in all countries, except that in Italy follow-up was based on linkage to a death register with no information on losses, and therefore analyses had to assume no losses to follow-up before the end date, and in France there were unknown numbers of losses to follow-up from emigration, in addition to a 4.1% known loss to follow-up.

Potentially, cancer and mortality risks in GH-treated patients could reflect the underlying condition leading to GH treatment, and the non-GH treatments given for this condition, as well as the effect of GH per se. One potential method to separate the effect of GH (if any) from that of the underlying diagnosis and its treatment would be to compare the GH-treated patients with other patients with the same condition who had not received GH. Such comparison data for untreated patients with the multiple underlying diagnoses involved in SAGhE in 8 countries do not exist, however, and anyway this would not entirely solve the problem, since selective factors leading to GH treatment may themselves cause differences in cancer risk between treated and untreated groups. We have therefore elected, as in the great majority of GH cohort studies [2,10,11,14,15,23,24,25,26,27], to use general population rates as the main comparator for rates in the GH patients. These comparison data will have the strength of stability through large numbers, and well-documented and understood data sources, but the analyses need, as in previous cohorts, to be interpreted carefully taking account that the comparison population did not have these diseases.

Finally, in principle, the source of the diagnosis of the cause of death should be the same for the cohort as for the comparison population. All SAGhE countries used national population mortality registry data, based on death certificate diagnoses, for ‘expected’ rates in the study cohort, but only five countries used solely the same source to identify cause of death in the GH-treated cohort; in France and Germany, clinical notes as well as death certificates were used, and in the Netherlands only clinical notes were used.

Overall, the uncertainties and weaknesses in the SAGhE data described above affect different countries for each facet of the data. Analyses and interpretation will therefore need to examine country-specific results as well as overall SAGhE results, and assess factors that might have influenced them (e.g. type of recruitment, extent of loss to follow-up), to check whether artefacts or biases might explain particular findings. We will analytically aggregate data from groups of countries, and exclude groups of countries with potential biases or artefacts in common, to determine whether this affects the results: for instance, we will analyse together countries with higher quality of follow-up or diagnostic accuracy, and examine the effect on results for patients treated for isolated growth failure of excluding countries for which, as noted above, regulations or reimbursement rules gave potential bias in recording this diagnosis. Since France constitutes 42% of the total cohort, we will examine the extent to which the results for SAGhE overall are consequent on French results.

The SAGhE cohort forms a major resource for investigating cancer and mortality risks in patients treated with r-hGH, now and in the future. With careful interpretation it should contribute greatly to determining whether this important treatment is safe.

## Statement of Ethics

In each country, appropriate ethics committee agreement was obtained. For all patients, either written informed consent was obtained, or an ethics committee agreed that consent was not required. The study complies with the ethical principles laid down in the Declaration of Helsinki.

## Disclosure Statement

K.A.-W. is a sponsor of several clinical GH trials supported by Pfizer and a recipient of investigator-initiated independent research grants from Pfizer, NovoNordisk, Ipsen and MerckSerono. P.C. is a consultant for MerckSerono. M.M. received lecture fees from Novo Nordisk and Lilly, and scientific meetings' travel grants from Ipsen, Novo-Nordisk, Pfizer and Sandoz. L.S. is a member of the NordiNet International Study Committee (Novo Nordisk) and a recipient of investigator-initiated independent research awards from MerckSerono (GGI award), NovoNordisk and Pfizer, as well as lecture honoraria from Ferring, Novo Nordisk, Pfizer and Merck Serono. J.-C.C. is an investigator in clinical trials using GH sponsored by Pfizer and by Lilly and in post-marketing studies using several brands of GH and received support for travel to international meetings from several GH manufacturers. S.C. has received lecture fees from Ipsen, Merck-Serono, Novo Nordisk and Pfizer, research grants from Merck Serono, Eli Lilly and Pfizer, support for travel to international meetings from several GH manufacturers and is member of PRISM advisory board (Ipsen). W.K. is a member of the Novo Nordisk Advisory Board on GH treatment and has received lecture and consultancy honoraria from Pfizer, Ipsen and Sandoz.

All other authors declare that they have no conflicts of interest.

## Supplementary Material

Supplementary data

## Figures and Tables

**Table 1:** SAGhE study: sources of identification of GH-treated patients

Country	Source of identification	Population-based^a^ or clinic-based	Identification (prospective^b^ or retrospective^c^)	Recording of GH patients on database	Estimated completeness of recruitment^d^
Belgium	National population-based register	Population-based	Prospective	Compulsory	98.4%

France	National population-basedregister	Population-based	Prospective	Compulsory	100%	

Germany	National industry database for 15 centres; clinical records as sole source for 1 centre and as additional source for 4 others (data in total from 16 of 70 known centres)	Clinic-based	Prospectively in industry database and retrospectively from clinicians	Voluntary	Leipzig, Magdeburg, Tübingen, Dresden, and Rosenheim: 100% Other centres: unknown, but recruitment entirely prospective

Italy	Regional databases (Piedmont and Campania), National Institute of Health Register (18 of 20 Italian regions), clinical notes from 2 centres in Rome	Partly populationbased (Piedmont and Campania and National Register); partly clinic-based	Prospective for databases Retrospective from clinical notes	Voluntary	Unknown, except Piedmont and Campania: 100% Rome: near 100%

The Netherlands	National population-based register	Populationbased	Prospective	Voluntary until 1997, compulsory after 1997	About 95% nationwide until 1997, 100% after 1997

Sweden	National population-based register	Populationbased	Prospective	Voluntary	99.5%

Switzerland	National registries for children with chronic renal failure and cancer Clinic databases and patient lists in 80% of Switzerland for all other diagnoses	Populationbased for renal failure and childhood cancer; clinicbased otherwise	Prospective for patients with renal failure and childhood cancer Retrospective as part of the SAGhE study for all other indications	Voluntary	Near to 100% of patients nationally with chronic renal failure and cancer; near to 100% for all other diagnoses in participating centres

*UK* England and Wales	National post-marketing surveillance study for all but 2 centres	Populationbased	Prospective	Voluntary	100% in some centres Others not known, but probably very high in post-marketing surveillance centres, less so in the 2 centres (of 21) using solely local registers
	Local clinical registries as sole source for 2 centres and as additional source for 2 others				
Scotland	National post-marketing surveillance study	Clinic-based (all clinics except Dundee)	Prospective	Voluntary	

a i.e. identified all subjects who live in a defined geographic area.

b i.e. recorded on a register at time of first GH treatment.

c e.g. identification made from existing case notes at the time of the SAGhE study.

d i.e. percentage of patients treated in this population/clinic who were identified for the study.

**Table 2:** SAGhE study: calendar periods of cohort entry and follow-up

Country	Years of starting GH treatment	Dates of mortality follow-up^a^	Dates of cancer incidence follow-up
Belgium	1985 – 2009	First GH treatment – December 31, 2010	January 1, 1999 – December 31, 2008 (Flemish region)

			January 1, 2004 – December 31, 2008 (Walloon and Brussels regions)

France	1985 – 1997	First GH treatment – September 21, 2009	January 1, 1985 – September 21, 2009

Germany	1985 – 2007	First GH treatment – February 28, 2010 (except Tübingen: August 31, 2011)	January 1, 1985 – February 28, 2010

Italy	1985 – 2008	January 1, 1999 – December 31, 2009	January 1, 1985 – June 20, 2010 or February 11, 2011, depending on when questionnaires for individuals were mailed

The Netherlands	1986 – 2007	First GH treatment – December 31, 2010	January 1, 1989 – December 31, 2011

Sweden	1985 – 1997	First GH treatment – December 31, 2010	January 1, 1985 – December 31, 2009

Switzerland	1986 – 2008	First GH treatment – December 31, 2010	January 1, 1985 – December 31, 2007, December 31, 2008, September 13, 2010, or December 31, 2011, depending on age and canton

UK	1984 – 2009	First GH treatment – September 30, 2013 (England and Wales)	January 1, 1984 – December 31, 2011 (England and Wales)
		First GH treatment – December 31, 2013 (Scotland)	January 1, 1984 – August 31 2011 (Scotland)

a Starting from the earliest GH treatment date (except Italy: January 1, 1999). The earliest use of r-hGH was in 1985 or 1986 in all countries except the UK, where the drug had first been used in trials in 1984.

**Table 3:** SAGhE study: sources of GH treatment data

Country	Source of GH data^a^	GH data collected prospectively^b^ or retrospectively^c^
Belgium	National clinically run database	Prospectively

France	Yearly forms sent by clinical centres to a national agency until 1997; hospital case notes thereafter	Prospectively until 1997; thereafter retrospectively from hospital case notes in 3 sweeps (in 1999, 2001 and 2010)

Germany	National industry database, clinical non-industry database, plus local clinic case notes	Prospectively in industry and clinical databases; clinic notes retrospectively

Italy	GH treatment registries (Piedmont and Campania); local clinic case notes and National Institute of Health Registry	National registry prospectively; clinic notes retrospectively

The Netherlands	Paediatric endocrinologists, hospital records, clinical notes	Prospectively, with missing data added retrospectively

Sweden	National database independent of industry	Prospectively in national database

Switzerland	Original medical charts at centres of paediatric endocrinology, oncology, and nephrology	Retrospectively

UK	National industry database plus local clinic case notes for a minority	Prospectively in industry database; retrospectively for hospital case notes

a i.e. doses, dates etc. for patients identified as GH-treated.

b i.e. at the time of treatment.

c i.e. extracted specially for SAGhE from existing case notes.

**Table 4:** SAGhE study: sources of follow-up information on vital status

Country	Source
Belgium	National population registry that also records deaths, emigrations and other exits

France	National civil status registry (RNIPP) and national health insurance registry (RNIAM)

No information on emigrations, but deaths abroad can be reported back to the French civil register

Germany	Municipal registries and health authority registries for each region that record deaths, emigrations and other exits

Italy	Deaths by linkage to national mortality database

The

Netherlands	National database that records current and previous addresses, deaths, emigrations and other exits

Sweden	National population register that records deaths, emigrations and other exits

Switzerland	Questionnaire responses, where obtained; failing that, municipal registers that record deaths, emigrations and other exits; failing that, last clinic visit

UK	National Health Service (NHS) Register that records deaths, emigrations and other exits

**Table 5:** SAGhE study: entry criteria and sources of follow-up information for cancer incidence analyses

Country	Criteria for entry to follow-up for cancer incidence	Source(s) of follow-up information on cancer incidence	Followed for cancer incidence, n
Belgium	All patients in the cohort	National cancer registration; questionnaires to patients; clinical notes	1,345

France	All patients except those with certain chronic diseases as the reason for GH treatment^a^	Questionnaires to patients, hospital insurance records that hold data on hospital discharges and medicines used	8,649
		Validation of diagnoses from hospital records when available; otherwise from coherence of diagnosis based on other sources	

Germany	Patients treated in Leipzig only	Questionnaires to patients, hospital records, and Leipzig cancer registry	559
		Validation of diagnoses from hospital pathology reports	

Italy	All patients in the cohort who replied to questionnaire	Questionnaires to patients No validation of diagnoses	737

The Netherlands	All patients in the cohort who did not decline to participate	National cancer registration	1,707

Sweden	All patients in the cohort who did not decline to participate	National cancer registration	2,832

Switzerland	All patients in the cohort	National childhood cancer registration for ages <16 or <21 years depending on the canton	745

UK	All patients successfully traced for follow-up at the NHS Central Registers	National cancer registration	3,902

Total			20,476

a Cancer, renal disease, syndromes with known high risk of cancer, acquired GH deficiencies, granulomatous diseases, total body irradiation, chemotherapy, bone marrow or solid organ transplantation.

**Table 6:** Cohort by sex, age, year of treatment, diagnosis, and country

	Belgium	France	Germany	Italy	The Netherlands	Sweden	Switzerland	UK	Total all countries
*Sex*									
Male	645 (46.7)	5,800 (56.2)	1,027 (57.6)	852 (62.5)	847 (47.9)	1,773 (59.8)	406 (54.1)	2,075 (53.2)	13,425 (55.4)
Female	737 (53.3)	4,516 (43.8)	757 (42.4)	512 (37.5)	921 (52.1)	1,192 (40.2)	345 (45.9)	1,827 (46.8)	10,807 (44.6)

*Age when GH treatment was started, years*									
0 – 4	144 (10.4)	704 (6.8)	116 (6.5)	33 (2.4)	191 (10.8)	310 (10.5)	72 (9.6)	451 (11.6)	2,021 (8.3)
5 – 9	382 (27.6)	3,114 (30.2)	553 (31.0)	327 (24.0)	624 (35.3)	1,099 (37.1)	266 (35.4)	1,395 (35.8)	7,760 (32.0)
10 – 14	716 (51.8)	5,428 (52.6)	957 (53.6)	886 (65.0)	788 (44.6)	1,366 (46.1)	363 (48.3)	1,756 (45.0)	12,260 (50.6)
15 – 19	140 (10.1)	1,070 (10.4)	158 (8.9)	118 (8.7)	165 (9.3)	190 (6.4)	50 (6.7)	300 (7.7)	2,191 (9.0)

*Year when GH treatment was started*									
<1990	407 (29.5)	2,998 (29.1)	131 (7.3)	14 (1.0)	232 (13.1)	664 (22.4)	21 (2.8)	831 (21.3)	5,298 (21.9)
1990 – 1994	402 (29.1)	5,709 (55.3)	522 (29.3)	140 (10.3)	699 (39.5)	1,563 (52.7)	181 (24.1)	1,271 (32.6)	10,487 (43.3)
1995 – 1999	278 (20.1)	1,609 (15.6)	676 (37.9)	638 (46.8)	568 (32.1)	738 (24.9)	252 (33.6)	1,110 (28.4)	5,869 (24.2)
≥2000	295 (21.3)	0 (0.0)	455 (25.5)	572 (41.9)	269 (15.2)	0 (0.0)	297 (39.5)	690 (17.7)	2,578 (10.6)

*Diagnosis leading to GH treatment^a^*								
CNS tumour and craniopharyngioma	208 (15.1)	768 (7.4)	141 (7.9)	33 (2.4)	231 (13.1)	219 (7.4)	60 (8.0)	667 (17.1)	2,327 (9.6)
Other solid tumour	15 (1.1)	41 (0.4)	10 (0.6)	1 (0.1)	18 (1.0)	0 (0.0)	11 (1.5)	65 (1.7)	161 (0.7)
Hematological malignancy	41 (3.0)	280 (2.7)	12 (0.7)	6 (0.4)	63 (3.6)	77 (2.6)	6 (0.8)	251 (6.4)	736 (3.0)
Chronic renal failure and renal diseases	7 (0.5)	130 (1.3)	15 (0.8)	13 (1.0)	8 (0.5)	32 (1.1)	42 (5.6)	66 (1.7)	313 (1.3)
Turner syndrome	348 (25.2)	1,382 (13.4)	287 (16.1)	94 (6.9)	328 (18.5)	301 (10.2)	70 (9.3)	694 (17.8)	3,503 (14.5)
Other syndromes and chronic diseases	135 (9.8)	285 (2.8)	153 (8.6)	49 (3.6)	170 (9.6)	191 (6.4)	112 (14.9)	450 (11.5)	1,545 (6.4)
Multiple pituitary hormone deficiency – organic GHD 153 (11.1)	884 (8.6)	220 (12.3)	30 (2.2)	328 (18.6)	269 (9.1)	100 (13.3)	513 (13.1)	2,497 (10.3)
Skeletal dysplasias	17 (1.2)	34 (0.3)	28 (1.6)	9 (0.7)	16 (0.9)	132 (4.5)	11 (1.5)	111 (2.8)	358 (1.5)
Isolated growth failure	458 (33.1)	6,476 (62.8)	913 (51.2)	1,109 (81.3)	585 (33.1)	1,545 (52.1)	333 (44.3)	1,049 (26.9)	12,468 (51.5)
Non-classifiable	0 (0.0)	36 (0.3)	5 (0.3)	20 (1.5)	22 (1.2)	199 (6.7)^b^	6 (0.8)	36 (0.9)	324 (1.3)
Total	1,382	10,316	1,784	1,364	1,768	2,965	751	3,902	24,232

Values are presented as numbers with percentages in parentheses. GHD = GH deficiency.

a Contents of the diagnostic categories are detailed in online supplementary table A1.

b More in Sweden than elsewhere mainly because of subjects whose diagnosis was known to the registry but could not be disclosed to the SAGhE study because of lack of patient consent.

**Table 7:** SAGhE study: numbers of patients identified and exclusions from the cohort for mortality follow-up

Country	Patients identified as treated with rGH	Clinics with incomplete retrospective recruitment of GH-treated patients	Date of birth or sex not known	Age ≥20 years at the start of GH treatment	Date when GH treatment started unknown	Date of end of follow-up not known	Follow-up not possible^3^	Total included in cohort for mortality follow-up
Belgium	1,389	0	0	5	0	2	0	1,382
France	10,332	0	0	6	0	10	0	10,316
Germany	1,839	19	0	1	29	6	0	1,784
Italy	3,853	0	4	5	9	0	2,471^a^	1,364
The Netherlands	1,770	0	0	2	0	0	0	1,768
Sweden	2,971	0	0	4	0	2	0	2,965
Switzerland	754	0	0	0	2	1	0	751
UK	4,642	0	0	3	17	0	720^a^	3,902

Total	27,550	19	4	26	57	21	3,191	24,232

a Italy, no follow-up beyond the start of the follow-up period (January 1, 1999); UK, lack of identifier data (n = 671) or of permission (n = 47) or otherwise could not be followed (n = 2).

**Table 8:** SAGhE study: losses to follow-up for mortality and cancer incidence

Country	Mortality				Cancer incidence			
	emigrated^a^	otherwise^a^ lost	total lost	lost to follow-up (not by death) before end date, %	emigrated	otherwise lost	total lost	lost to follow-up (not by death or cancer) before end date, %
Belgium	32	4	36	2.6	18	0	18	1.3
France	_b	421	421	4.1^b^	–	–	_^c^	_^c^
Germany	1	78	79	4.4	0	4	4	0.7
Italy	–	–	_^c^	_b	–	–	_^c^	_^c^
The Netherlands	38	19	57	3.2	38	19	57	3.3
Sweden	37	0	37	1.2	36	0	36	1.3
Switzerland	28	8	36	4.8	11	2	13	1.7
UK	31	53	84	2.2	20	35	55	1.4
Total^d^	167	162	750	3.3	123	60	183	1.7

Values presented are numbers unless otherwise stated.

a Numbers attributed to emigration versus other reasons for loss to follow-up will depend on the quality of data available on reason for loss, as well as the extent of losses for different reasons.

b No data available on emigrations, which are additional to the losses shown.

c No data available on losses to follow-up.

d Excluding Italy for mortality, and excluding Italy and France for cancer incidence.
