# Supplementary material for: Description of the SAGhE Cohort: A Large European Study of Mortality and Cancer Incidence Risks after Childhood Treatment with Recombinant Growth Hormone
Source: Horm Res Paediatr. 2015 Jul 23;84(3):172–83. doi: 10.1159/000435856 (PMC4611066; doi:10.1159/000435856)
Supplement: Supplementary file 1 — Supplementary data [file hrp-0084-0172-s01.pdf]

Table A1: Diagnostic classification

**Diagnostic group, abbreviation**	**Diagnoses**	**Hormonal status and mechanism for growth failure**
Central nervous system tumours, CNST	Mostly medulloblastomas / primitive neuroectodermal tumors, ependymomas, gliomas, and germ cell tumours	Most of these patients have severe growth hormone deficiency due to the tumour or to radiotherapy to the hypothalamic-pituitary area
Craniopharyngioma, CRA	Craniopharyngioma	These patients have almost invariably severe and multiple pituitary hormonal deficiencies due to the tumour, surgery and radiotherapy
Childhood solid tumours affecting other organs than the CNS, NCNST	Small and heterogeneous group of patients mostly composed of facial tumours with irradiation involving the hypothalamic-pituitary area	Those who have received irradiation on the hypothalamic-pituitary area generally have growth hormone deficiency; the mechanism for growth retardation is less clear in some of the other cases
Haematological malignancies, HM	Mostly leukaemias and lymphomas; also includes bone marrow transplantation for leukaemia	Growth hormone deficiency after prophylactic craniospinal irradiation or total body irradiation; in addition, growth can be altered by other mechanisms (direct radiation-induced damage to the spine; toxic effect of chemotherapy to the growth plate)
Chronic renal failure and chronic renal diseases, CRF	Mostly chronic renal failure, tubular disorders (Fanconi syndrome) and to a lesser extent kidney transplantation	Normal growth hormone secretion; alterations of the growth hormone / IGF-1 system due to chronic renal failure
Turner syndrome, TS	Turner syndrome (loss of all or part of the second sex chromosome) with various chromosomal complements	Normal growth hormone secretion; growth failure partly due to loss of the SHOX gene on the short arm of chromosome X
Other syndromes and chronic childhood diseases, SCD	Very heterogeneous group composed of congenital syndromes (mostly Noonan, Prader-Willi, Silver-Russel and neurofibromatosis type 1 without CNS tumour) and chronic childhood diseases (mostly chronic anaemia, cystic fibrosis, Crohn disease, juvenile arthritis)	Normal growth hormone secretion; various mechanisms involved
Multiple pituitary hormonal deficiencies and organic growth hormone deficiency, MPHD/OGHD	Growth hormone deficiency associated with a hypothalamic-pituitary MRI abnormality (in particular ectopic posterior pituitary) or associated with other pituitary hormone deficiencies; of note the diagnosis of other hormonal deficiencies is not always straightforward and the hormonal situation can fluctuate; any indication for associated hormonal deficiency (i.e. associated treatment) was considered as such in the classification	Growth hormone deficiency is usually severe and permanent (persisting in adulthood) when it is associated with MRI abnormalities or other pituitary hormonal deficiencies
Skeletal dysplasia, SKD	Mostly hypochondroplasia, Leri-Weill dyschondrosteosis and non classified skeletal dysplasia; patients with achondroplasia are also included in this group	Normal growth hormone secretion; short stature is due to growth plate abnormalities
Isolated growth failure, IGF	Predominant group with short stature associated with either prenatal growth failure (children born SGA), failed growth hormone stimulation tests relative to cut-offs in use or constitutional or idiopathic short stature	Variable degree of growth hormone impairment as described by the peak growth hormone value after stimulation; the degree of constitutional growth impairment is reflected by birth length and weight and by target height (mid-parental height); all these variables are included in the multivariate analyses
Non-classifiable, NC	Insufficient or inconsistent information to allow a valid classification	

.

Table A2: Risk group classification

**Risk groups**	**Diagnostic groups (see table #)**	**Expected mortality risk**	**Comment**
Risk group 1a	Patients with IGF, some patients with OGHD, some patients with SKD,not known to have been born small for gestational age[1]	This group is expected to have a similar mortality risk relative to the general population	Isolated growth hormone deficiency, whether idiopathic or acquired (trauma, neonatal causes) or associated with minor craniofacial malformations (such as cleft lip) or ectopic posterior pituitary (if no associated hormonal deficiency)<br>Mild skeletal dysplasia (hypochondroplasia, dyschondrosteosis)<br>Familial and idiopathic short stature<br>Constitutional delay of growth and puberty
Risk group 1b	Patients with IGF, OGHD, SKD, known to have been born small for gestational age[1]	Smallness at birth is associated with increased cardiovascular risk in adulthood	All patients in group 1 who are defined as SGA irrespective of any coexisting isolated growth hormone deficiency
Risk group 2	TS, SCD, MPHD, most patients with OGHD, some patients with SKD	This group is expected to have an increased mortality risk relative to the general population, based on common knowledge or published evidence	All patients with clinically defined syndromes, including Turner, Noonan, Prader-Willi, Silver-Russel and neurofibromatosis type 1 (in the absence of CNS tumour)<br>Short stature or growth hormone deficiency associated with severe cerebral or extra-cerebral malformation<br>Severe chronic paediatric diseases such as chronic intestinal inflammatory diseases, metabolic diseases, mitochondrial disorders, chronic anaemia (sickle cell disease, thalassemia major), chronic inflammatory diseases (lupus, juvenile arthritis), diabetes<br>Multiple pituitary hormone deficiency, whatever the associated hormonal deficiency and the timing of appearance of the second pituitary hormone deficiency<br>Benign pituitary lesions other than craniopharyngioma (adenomas, cysts)<br>Severe skeletal dysplasia (achondroplasia, osteogenesis imperfecta, spondyloepiphyseal dysplasia)
Risk group 3	CNST, CRA, NCNST, HM, CRF<br>Some patients of the SCD group	This group is expected to have a markedly increased mortality risk, relative to the general population, based on common knowledge and published evidence	All patients with intra- or extra-cerebral malignancies, including Langerhans cell histiocytosis and patients having received a bone marrow or solid transplantation for a non-malignant disease<br>Syndromes with known increased risk for malignancies: Bloom, Fanconi, Down,and chromosomal breakage syndromes

[1]Small for gestational age defined as birth weight and/or birth length <2SDS, based on country-specific reference data.

The SAGhE cohort: a large European study of mortality and cancer incidence risks after childhood treatment with recombinant growth hormone

Supplementary Material

Because the initial diagnosis for which patients receive r-hGH heavily influences their future mortality and other outcomes, and there were many different initial diagnoses, often with few cases per diagnosis, we devised eleven categories of initial diagnosis (Table A1) and four larger mortality-risk based groupings (Table A2), which we used in analyses requiring larger numbers. The latter grouping was created based on the current literature and clinical judgment about the prognosis of the individual diagnoses.

To categorise individuals into the groups, we used the data on diagnosis and birth characteristics that we had extracted from existing databases and case-notes, and used them to assign for each patient up to five diagnostic codes using the European Society for Paediatric Endocrinology (ESPE) classification of paediatric endocrine diagnoses [12] and the international classification of childhood cancer [13]. In addition, associated pituitary hormonal treatments (thyroid hormones, glucocorticoids, sex steroids or gonadotropins, desmopressin) and karyotype results were compiled. Birth characteristics were classified as small for gestational age (SGA) if birth length or weight were below -2 SD for the gestational age. Each patient was then individually assigned to one of the diagnostic categories (Table A1) and risk group categories (Table A2) combining all the available information. Diagnostic and risk group categorization was done centrally and blind to the mortality or other study outcomes, based on the information provided by each country.

Reference List

12. Wit J, Ranke M, Kelnar CJH. ESPE classification of paediatric endocrine diagnoses. Horm Res. 2007;**68** (suppl.2):I-iX

13. Steliarova-Foucher E, Stiller C, Lacour B, Kaatsch P. International Classification of Childhood Cancer, third edition. Cancer. 2005;**103**:1457-67
